# National survey on pediatric respiratory physiotherapy units: primary ciliary dyskinesia and non-CF bronchiectasis

**DOI:** 10.1186/s13052-025-01904-0

**Published:** 2025-03-06

**Authors:** Beatrice Tani, Nicola Ullmann, Paola Leone, Alessandra Boni, Eugenio Barbieri, Matteo D’Angelo, Sara De Dominicis, Beniamino Giacomodonato, Stefania Monduzzi, Irene Piermarini, Chiara Pizziconi, Beatrice Ferrari, Renato Cutrera

**Affiliations:** 1 Pediatric Pulmonology & Cystic Fibrosis Unit, Bambino Gesù Children’s Hospital, IRCCS, Rome, Italy; 2 Rehabilitation Unit, Meyer Children’s Hospital IRCCS, Florence, Italy

**Keywords:** Primary Ciliary Dyskinesia, Non-Cystic Fibrosis bronchiectasis, Pediatrics, Physiotherapy unit, Respiratory physiotherapy, Survey, Physiotherapy procedures, Airway clearance

## Abstract

**Background:**

Currently, there is a lack of data concerning the organization and characteristics of Italian pediatric physiotherapy units for the treatment of patients with chronic lung diseases, especially those with rare conditions such as Primary Ciliary Dyskinesia (PCD) and non-Cystic Fibrosis bronchiectasis (NCFB).

**Methods:**

A national descriptive study based on a survey questionnaire was conducted. The questionnaire consisted of three different sections: distribution and characteristics of the centres, services provided by respiratory therapists, physiotherapists’ perception of the unit. The survey was distributed to all healthcare providers via an online platform, and a descriptive data analysis was performed.

**Results:**

The survey had a response rate of 97.5% with twenty-nine responses collected. The centers are heterogeneously distributed: thirteen in the northern regions, eight in the central regions and eight in the southern regions. Of the 29 centers with a physiotherapy unit, 19 had a specialized respiratory therapy unit. Respiratory therapy was provided in different care settings: regular wards (28/29 centers, 97%), outpatient service (29/29 centers, 100%), and intensive or semi-intensive care units (17/29 centers, 59%). The interventions provided by respiratory therapists involved more than just airway clearance (29/29). More specific interventions, such as pulmonary function tests (23/29), functional tests (27/29), educational training (26/29), management of workout exercise programs (25/29) and interventions developed in collaboration with physicians such as non-invasive ventilation (NIV) (23/29) and oxygen titration (21/29) are performed. It is interesting to note that therapists are also involved in various activities, such as telemedicine, physiotherapists’ research projects, and supporting alongside physicians, for the prescription at home of medical devices. Perception of the unit was also evaluated.

**Conclusions:**

The involved centers are heterogeneous in terms of distribution and treatments offered. The role of respiratory physiotherapists still seems to be fragmented. This first descriptive analysis of the physiotherapy units and the main differences between centers opens queries on the clinical approaches used for pediatric patients with PCD in terms of respiratory physiotherapy. However,in response to evolving treatment needs, a more specialized and standardized approach to patient care is required.

## Background

PCD and NCFB are two rare conditions affecting the respiratory system. PCD is a genetic disorder of motile cilia, primarily transmitted as an autosomal recessive trait [1]. Motile cilia are located on the apical surface of the upper and lower respiratory tract, on the ependymal cells lining the ventricles of the central nervous system, as well as on the oviducts of the female reproductive system and in the flagellum of male spermatozoa [2]. The clinical phenotype of PCD is broad but respiratory manifestations are part of the typical presentation of the disease and are central in the management and treatment of patients [3].

On the other hand, NCFB has been recognized as a clinical problem in children for approximately two centuries [4] and is characterized by progressive and often irreversible bronchial dilatation due to structural changes in the bronchial wall and chronic airway inflammation [5]. NCFB is a heterogeneous disorder and recognizes several underlying etiologies, including PCD, which has been suggested in various studies to be the primary cause of its onset [6–8].

Both conditions are characterized by daily productive chronic cough [5] and recurrent upper and lower respiratory tract infections [3, 9].

To date, chest physiotherapy is one of the cornerstones of treatment and prevention of exacerbations and management of bronchial secretions [10, 11]. Treatment should be initiated in early childhood [12]. Currently, therapeutic strategies for PCD and NCFB do not base on validated disease-specific recommendations, but often refer to the available evidence for Cystic Fibrosis (CF) [3, 13]. Physiotherapy treatments should follow common protocols and the approaches offered should not differ between centers. To the best of our knowledge, no data have been published on the organization of physiotherapy units for pediatric patients with PCD and NCFB in Italy and neither in European territory.

The primary objective of this survey was to identify Italian pediatric centers with a respiratory therapy team and to determine their characteristics and the services offered, with the aim of obtaining a primary overview of the Italian context and set up of a professional network.

## Materials and methods

A multidisciplinary team, consisting of respiratory therapists and pediatric respiratory physicians, designed a survey questionnaire to be distributed to Italian pediatric centers treating PCD and NCFB. Given that the Italian National Healthcare system does not currently centralize care for PCD and NCFB [14], we reached out the major number of CF centers, children’s hospitals, and general hospitals for our study.

The questionnaire was designed to assess the services provided in Italian healthcare providers in relation to respiratory therapy. In order to involve as many centres as possible, we contacted all the members of the SIMRI (Italian society of pediatric respiratory diseases) study group “PCD and NCFB” and the physiotherapy group of the SIFC (Cystic Fibrosis Italian Society). We assume that the interviewed centers truly represent the major healtcare providers that manage this patient population, given that these two association are key national Society for rare pediatric respiratory diseases in Italy.

Centers that did not treat pediatric patients with PCD or NCFB were excluded from the survey.

Clinicians who declared not to have a physiotherapy service at their centers, were excluded from the survey and were subsequently contacted to inquire about the patients’ physiotherapy management with a shorter set of questions sent by email.

The questionnaire was sent via an online survey platform to the physiotherapist head of department (or delegate) of each unit. One week before the survey was distributed, an email was sent to each participant explaining the purpose of the survey. The survey was sent out in May 2023, and responses were collected until August 2023. Centers were prompted through a weekly email reminder.

### Questionnaire

We investigated various aspects related to the organization of physiotherapy units. Specifically, the questionnaire was divided in three sections:

1. Distribution and characteristics of the centers.
2. Services provided by respiratory therapists.
3. Physiotherapists’ perception of the unit.

#### Distribution and characteristics of the centers

To obtain a national overview of the centers, we first identified the location of each hospital and the corresponding Italian region.

Next, we asked for detailed information on the total number and age of the patients with NCFB and PCD followed in each unit.

#### Services provided by respiratory therapists

The second section of the questionnaire investigated the physiotherapy unit. In particular, the team composition, the existence of a specific respiratory physiotherapy unit, the tasks of the physiotherapists and the settings of intervention. Finally, we inquired the instrumental and functional tests performed by the team.

#### Physiotherapists’ perception of the unit

Physiotherapists’ perception was investigated in two different areas. First, team’s skill in airway clearance was assessed. Five different airway clearance techniques were analyzed and a Likert scale (No skills – Highly specialized) was used to determine the perceived mastery. Secondly, the availability of instruments and devices was assessed.

### Analysis

This descriptive study was based on data obtained from the participating centers’ questionnaires. Once all available responses were collected, data were entered into a database for analysis. All data analysis was performed by the principal investigator and shared with other researchers. Data were collected in a database and statistical analysis and graphs processing were performed using Excel (16.0 Software). Response rate was calculated taking into account responders and missing in relation to the involved centers. Absolute frequency and percentage were calculated for categorical variables, and the mean value was calculated for continuous variables. For open-ended questions, responses were categorized and analyzed narratively. To measure liking of the procedure a 5-point Likert scale was used.

Since the survey included only general information and did not disclose any sensitive patient data, formal approval from the Ethical Committee was not sought.

## Results

**Fig. 1:**
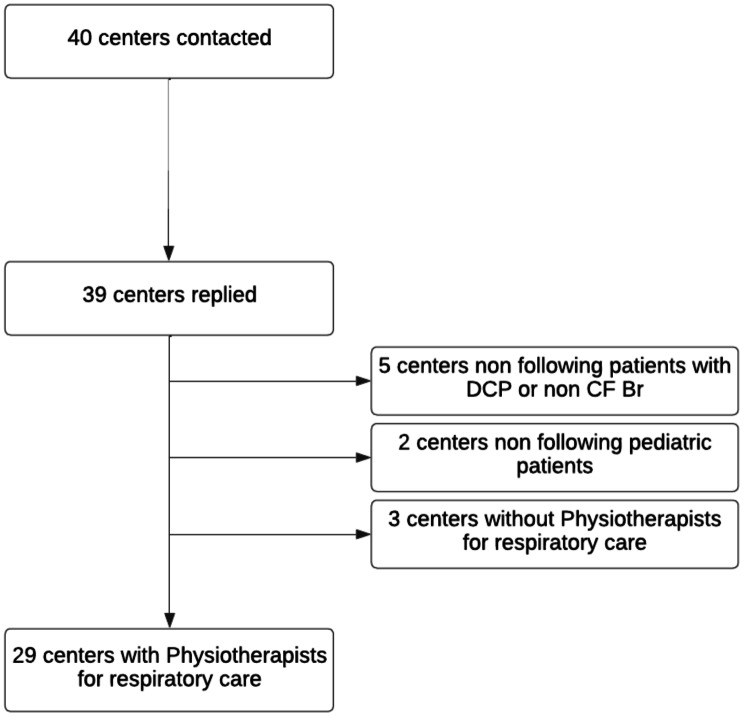
Flowchart of “National Survey on Pediatric Respiratory Physiotherapy Units: Primary Ciliary Dyskinesia and Non-CF Bronchiectasis” sent to Italian Pediatric respiratory units treating PCD and NCFB pts

As shown in Fig. 1, out of 40 centers contacted, 39 replied (97.5%). Ten centers were excluded from the survey: seven reported not to treat pediatric patients with PCD or NCFB and three centers reported not have physiotherapy unit/physiotherapists for respiratory care. To these centers was provided a shorter set of questions about the physiotherapy management for their patients, with responses provided by the contacted physician. For the three centers, patient care was delegated to other facilities outside the region of residence (*n* = 1), to nurses (*n* = 1) and to physicians only for techniques review (*n* = 1).

### Distribution on the territory and centers’ characteristics

**Fig. 2:**
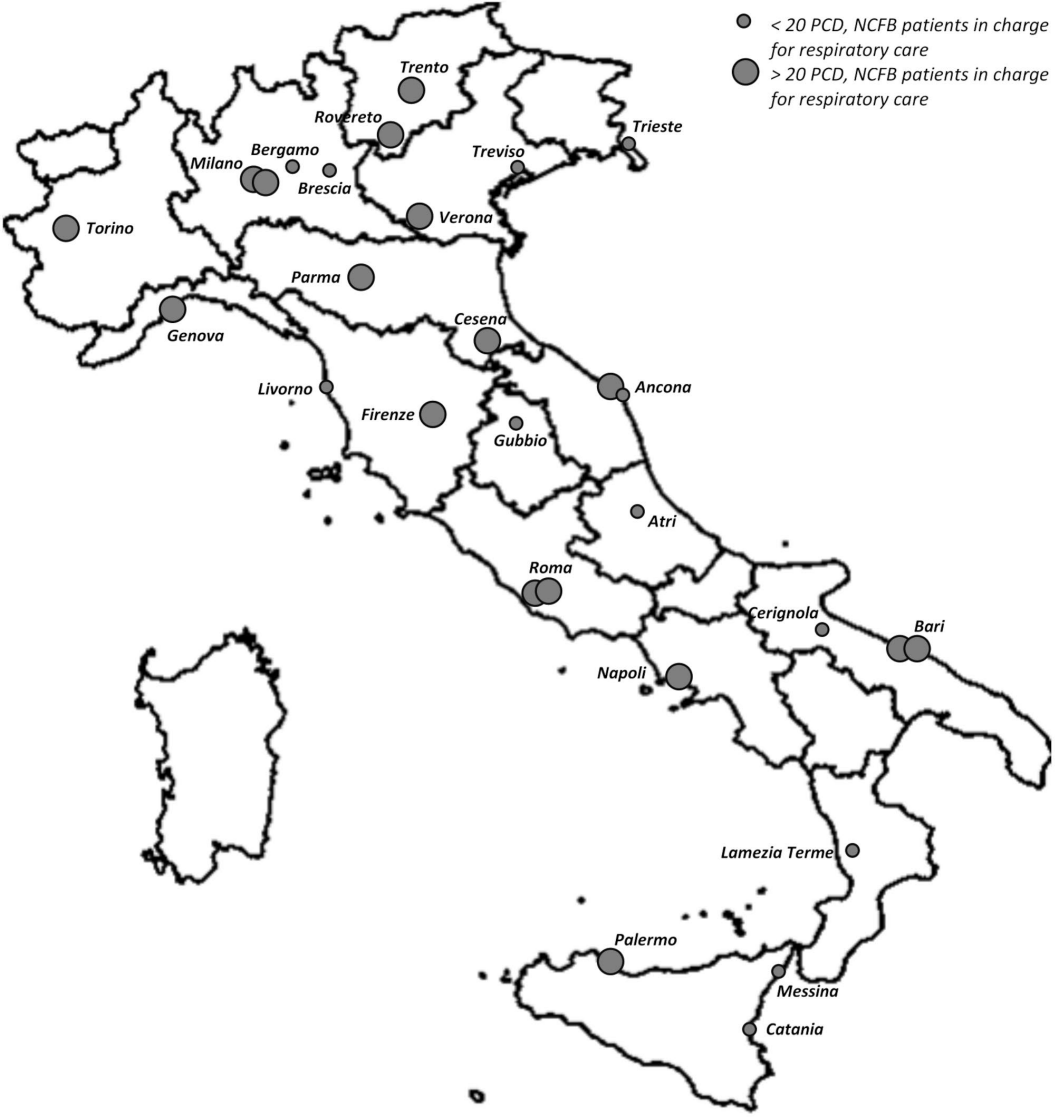
Geographical representation of centers with respiratory therapy unit or respiratory therapists treating PCD and NCFB pts. North: Valle d’Aosta, Piemonte, Liguria, Lombardia, Trentino-Alto Adige, Veneto, Friuli-Venezia Giulia, Emilia Romagna; Center: Toscana, Marche, Umbria, Lazio, Abruzzo, Molise, Sardegna; South: Campania, Puglia, Basilicata, Calabria, Sicilia

The distribution of centers with a physiotherapy unit for respiratory care that completed the survey, and the relative number of PCD and NCFB patients managed is represented in Fig. 2. We managed to reach centers from the north to the south of Italy and showed a heterogeneous distribution of centers throughout the country. Four Italian regions were not represented (3 did not retrieved, 1 reported not having a center with a respiratory physiotherapy unit). Thirteen centers are located in the north Italian area, 8 in the center and 8 in the south. Among the included centers, 25/29 (86,2%) took in charge also CF patients, and 15/29 centers are regional referral centers for Cystic Fibrosis. Given this purpose, even rare conditions such as PCD and NCFB can benefit from the service and the specific expertise of its professionals.

Regarding the age of patients took in charge, adults centers treating pediatric population, represented the majority of centers: 25/29 of the interviewed. Out of these 25 centers, 17/25 treated mostly adult patients, 8/25 mostly paediatric patients (0–18 y.o.).

### Services provided by respiratory therapists

Regarding the organization of physiotherapy team, many differences were noted. Of the 29 centers with a physiotherapy unit, only 19 had a specialized respiratory therapy unit, whereas 10 offered a general physiotherapy service. Of these 10 units, 4 reported spending < 50% of their daily time on respiratory care, whereas 6 reported spending ≥ 50% of their daily time on the respiratory care.

The centers had an average of 4.1 professionals per unit in the north, 3.4 in the center, 1.8 in south.

Respiratory therapy intervention was administered in different care settings: regular ward (28/29 center, 97%), outpatients (29/29 center, 100%), intensive or semi-intensive care setting (17/29 center, 59%).

**Fig. 3:**
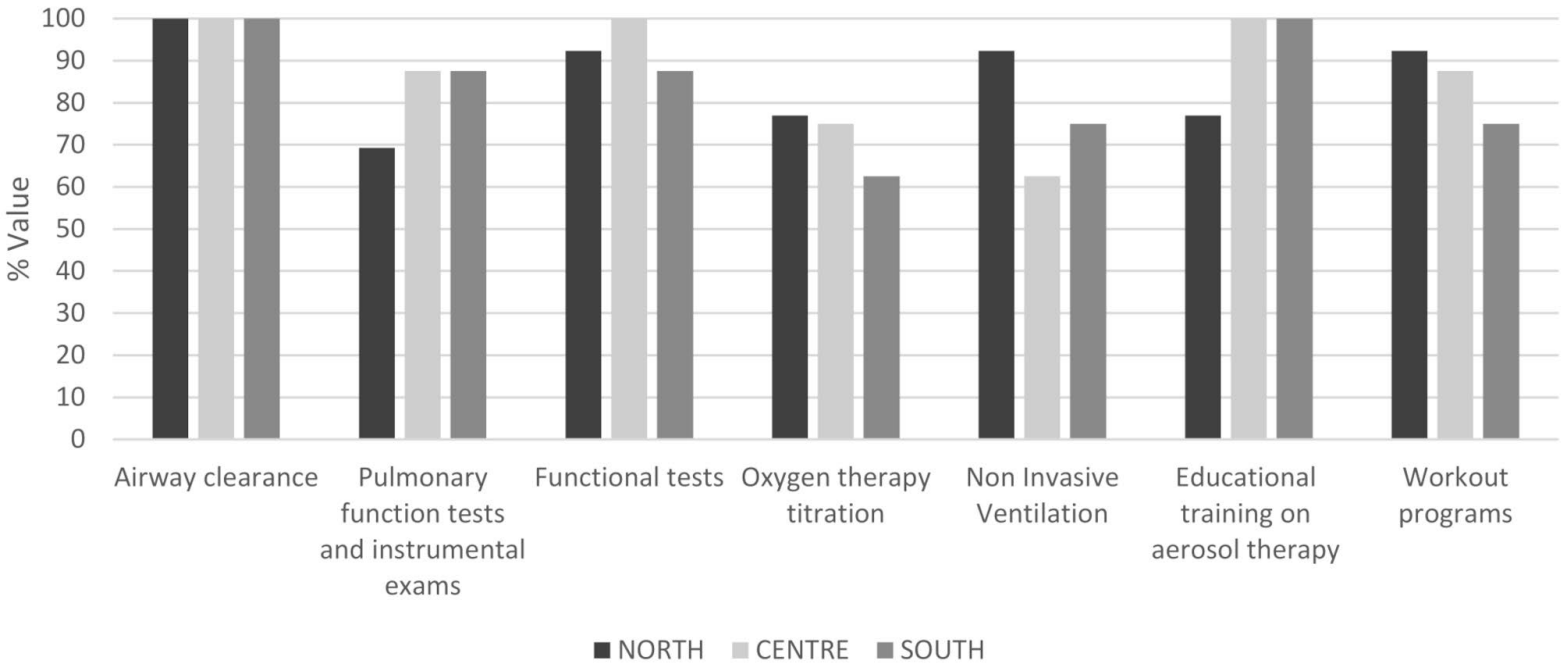
Services offered. Service offered from respiratory therapists in general admission patients - % centers

The interventions performed by respiratory therapists are shown in Fig. 3. These interventions include more than just airway clearance, which is the common element for the centers (29/29, 100%). Physiotherapists performed more specific interventions such as pulmonary function tests (such as spirometry, DLCO, etc.) (23/29, 79,3%), functional tests (such as 6MWT, SPPB test, etc.) (27/29, 93,1%), educational training (26/29, 89,6%), management of workout programs (25/29, 86,2%) and intervention developed in collaboration with physicians, as NIV (23/29, 79,3%) and oxygen titration (21/29, 72,4%).

According to tests and procedures performed by physiotherapists, major geographical differences were found in the hospital ward activities. In addition to airway clearance techniques (100% north, center, south), units are usual to perform mostly pulmonary function tests (84.6% north, 87,5% center, 100% south) and functional tests (92,3% north, 100% center, 100% south) in outpatient regimen, conversely oxygen titration (69,2% north, 62,5% center, 12,5% south), NIV titration (92,3% north, 62,5% center, 12,5% south) and the set-up of workout programs (69,2% north, 87,5% center, 50% south) are performed mainly in hospital wards.

Table 1 summarizes the tests and procedures more frequently performed by therapists both outpatient setting and hospitalization care.

**Table 1:** Tests and procedures performed by physiotherapists

%	PROCEDURES PERFORMED
82	Six minute walking test (6MWT)
71	Spirometry
29	Sit to stand test
21	Bronchial provocation test and bronchial dilation test
	Measurement of maximal inspiratory and maximal expiratory (MIP/MEP) pressures
	Medical Research Council (MRC) scale
	Shuttle walking test (MSWT)
18	Multi-breath washout test
	Sub-Maximal Cycle Ergometer & step Test
14	Body plethysmography
	Fractional exhaled nitric oxide test (Feno)
11	Diffusing Capacity of the Lungs Test (DLCO)
	Impulse oscillometry (IOS)
	Nocturnal pulse oximetry
	Peak Expiratory /Inspiratory Flow (PEF/PIF), Peak expiratory cough flow (PCEF)
	MultiBreath Washout test
	Short Physical Performance Battery (SPPB scale)
7	Chest ultrasound
4	Challenge test
	Tidal Breathing Pulmonary Function of Children < 2 y.o.
	Forced oscillation technique (FOT)
	Indirect calorimetry
	Cardiopulmonary exercise testing (CPET)
	Bruce modified test
	One-repetition maximum test (1RM)

Moreover, we also asked to describe additional services provided by therapists in each center. Therapists were also involved in different activities such as telemedicine, research projects, and in the support, alongside physicians, for prescription at home of medical devices. Additionally, the collection of biological material for microbiological examination is often entrusted to respiratory therapists.

### Perception of physiotherapists of the unit

**Fig. 4:**
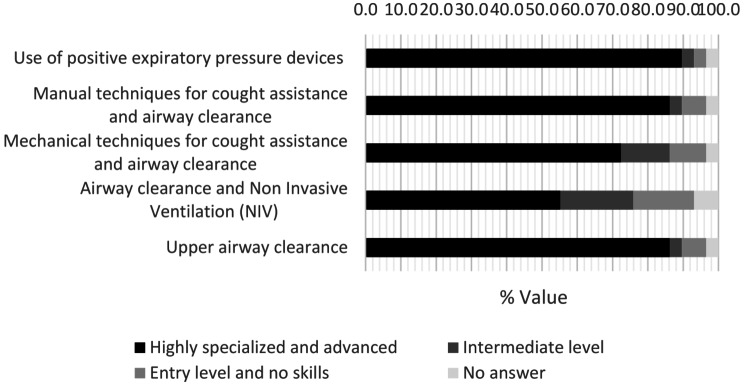
Perceived mastery of airway clearance techniques

Figure 4 represent the self-assessment Likert scale of team’s skill in five different airway clearance techniques. Secondly, concerning physiotherapists’ perception of the units’ devices availability, 17% of the therapists consider it inadequate.

## Discussion

This study provides for the first time an overall view of the national distribution, organization, and structure of physiotherapy departments following pediatric patients affected by PCD and NCFB. To achieve this objective, the study was conducted with the collaboration of a great number of centres that are representative of the whole Italian territory.

Previous surveys on physiotherapy have mainly focused on techniques and clinical management of patients [10, 15], and more specific data on the Italian physiotherapy units is lacking.

The importance of services provided by hospital-based physiotherapy departments is increasingly recognized, particularly in the multidisciplinary treatment of respiratory diseases. This need is even greater for rare conditions as PCD and NCFB, which, as has been reported in the literature over the last decade [10, 16] require the establishment of daily airway clearance treatment and individualized exercise programs to reduce the decline of lung function over time. For this reason, it seemed appropriate to study the distribution of physiotherapy units on the Italian territory that follow pediatric PCD and NCFB patients, and then try to identify the organization and services offered by each center.

In our survey we achieved an excellent response rate of 97.5%, confirming the positive attitude and increasing interest of Italian physiotherapists to be engaged in research projects. Unfortunately, we are missing data from three regions (Valle D’Aosta, Molise, Basilicata) in our survey. As hypothesized in our previous national research project [14], this can be related to the absence of specialized centers in those regions or to our difficulties in reaching all the territorial centers. Moreover, the three centers lacking a physiotherapy unit for respiratory care, declared to refer patients to facilities outside the region or to medical/nurse care. Our data reveals the challenge in finding professional physiotherapists trained in respiratory care. We all agree that a well-planned chest therapy program is essential, and the competencies that physiotherapists are acquiring over time are of paramount importance, as suggested by the literature [3, 17]. These data, highlighting the potential absence of professional respiratory therapists in some Italian regions should be seriously taken into account.

Analyzing the distribution and features of the centers, the primary distinction lies is in terms of territorial distribution, particularly between the northern (13 centers) versus center/southern Italian area (8 centers/8 centers). The heterogeneity of units is evident, and this diversity extends to the organization of logistic teams. Respiratory therapy predominantly takes place in outpatient and regular ward settings as observed in 100% and 96,5% of centers respectively. In contrast, intensive and semi-intensive care units play a partial role in the work of physiotherapists treating PCD and NCFB, with interventions being conducted in only 17 out of 29 centers. Furthermore, concerning team composition, among the 29 centers, 19 (65%) have a dedicated respiratory team. Nevertheless, therapists still spend ≥ 50% of their time on respiratory care in 6 out of 10 units. This leads us to hypothesize that the need for respiratory physiotherapy is definitively growing due to a progressively greater number of diagnosed rare respiratory diseases or an increasing importance given to respiratory physiotherapy treatments.

In our survey we retrieved a decreasing average number of respiratory therapists per center, going from the north to the south region of our country. We could hypotize that the northern centers have a greater availability of respiratory therapists. However, we cannot show if this data is related to a major workload of patients treated from the unit.

Considering the interventions made by respiratory therapists, our survey showed that professionals not only play an irreplaceable role in developing an airway clearance program. In fact, assessment of patient condition through tests and pulmonary function tests, educational training for caregivers, titration of oxygen definition and NIV, and workout programs are usual care activities in which Italian respiratory therapists are involved. This highlights the crucial role of respiratory therapists on multidisciplinary teams dealing with PCD and NCFB [18]. Moreover, we appreciate how physiotherapists are expanding their expertise in respiratory care, and the growing competence of these professionals is aligning with the increasing knowledge and consideration of these rare diseases.

Additionally, our survey revealed heterogeneity between healthcare providers in the interventions provided by physiotherapists, possibly due to the unique needs and specificities of each center. In the majority of centers, therapists perform walking tests and spirometry. However, it is important to note that in almost 20% of centers, tests such as bronchial provocation and dilation tests and Multi-Breath Washout tests, are also conducted by physiotherapy units. Definitively fewer centers perform more specialized procedures such as DLCO, FOT and IOS. These results show that according to the specific needs of centers, physiotherapists are professional figures that can perform various respiratory tests and functional evaluations.

Related to the professional perception of the offered service, our study emphasized that although airway clearance is a core element in the treatment of PCD and non NCFB, there is still a varying perception of the confidence of the different airway clearance techniques. In many cases, therapists do not feel fully confident when using more complex techniques, such as airway clearance using instrumentation (as IPV, cough machine, EFA and HFCWO) and with NIV. Moreover, the adequacy of devices available was considered inadequate in 17% of units. This result strongly highlights the importance of addressing the training needs of professionals involved in the respiratory field and further implementing the devices needed in all centers. This could ensure professionals and patients to have the same clinical possibilities for high quality treatment.

Finally, our results raise new questions about the evolution of respiratory therapists’ skills. These include telemedicine, chest ultrasounds and collaboration with physicians in the titration and training process for NIV and oxygen therapy. This evolution could be explained by the need for a more specialized approach to patient care. Establishing a network of professionals throughout the national territory is crucial to address the new challenge that the future presents, namely the increase in complex cases and, consequently, in healthcare needs.

## Conclusions

In conclusion, this national survey showed for the first time discrepancies in the national distribution of the physiotherapy units involved in respiratory management of PCD and NCFB patients. Moreover, we revealed that respiratory physiotherapists in Italy perform various specialized techniques not only related to airway clearance, depending on the needs of each center. Finally, our work allowed us to define the characteristics and the growing expertise of the therapists included in respiratory teams and marks the first step in fostering cooperation among units and in the standardization on the approach to patient care.

## Data Availability

The datasets used and/or analysed during the current study are available from the corresponding author on reasonable request.

## Abbreviations

PCD: Primary ciliary dyskinesia
NCFB: Non-Cystic Fibrosis Bronchiectasis
NIV: Non-Invasive Ventilation
DLCO: Diffusing Capacity of the Lungs for carbon monoxide Test
FOT: Forced oscillation technique
IOS: Impulse oscillometry
IPV: Intrapulmonary Percussive Ventilation
EFA: Expiratory Flow Accelerator
HFCWO: High-frequency chest wall oscillation
Pts: Patients
